# Short-term effectiveness of transcranial direct current stimulation in the nociceptive behavior of neuropathic pain rats in development

**DOI:** 10.3934/Neuroscience.2023032

**Published:** 2023-12-15

**Authors:** Priscila Centeno Crespo, Leo Anderson Meira Martins, Otávio Garcia Martins, Clara Camacho Dos Reis, Ricardo Netto Goulart, Andressa de Souza, Liciane Fernandes Medeiros, Vanessa Leal Scarabelot, Giovana Duzzo Gamaro, Sabrina Pereira Silva, Marcos Roberto de Oliveira, Iraci Lucena da Silva Torres, Izabel Cristina Custódio de Souza

**Affiliations:** 1 Postgraduate Program in Biochemistry and Bioprospection, Universidade Federal de Pelotas, Pelotas (UFPel), Pelotas, Rio Grande do Sul (RS), Brazil; 2 Laboratory of Cellular Neuromodulation: Basic Sciences, Institute of Biology, Department of Morphology, UFPel, Pelotas, RS, Brazil; 3 Department of Physiology, Institute of Basic Health Sciences, Universidade Federal do Rio Grande do Sul (UFRGS), Porto Alegre, RS, Brazil; 4 Laboratory of Pain Pharmacology and Neuromodulation: Preclinical Researches, Hospital de Clínicas de Porto Alegre (HCPA), Porto Alegre, RS, Brazil; 5 Postgraduate Program in Health and Human Development, Universidade La Salle, Canoas, RS, Brazil; 6 Postgraduate Program in Medicine Medical Sciences, Medicine School, UFRGS, Porto Alegre, RS, Brazil; 7 Department of Biochemistry, UFRGS, Porto Alegre, RS, Brazil

**Keywords:** brain derived neurotrophic factor, development, neuropathic pain, noninvasive brain stimulation, transcranial direct current stimulation

## Abstract

Neuropathic pain (NP) is caused by a lesion that triggers pain chronification and central sensitization and it can develop in a different manner, dependent of age. Recent studies have demonstrated the efficacy of transcranial direct current stimulation (tDCS) for treating NP. Then, we aimed to investigate the effects of tDCS and BDNF levels in neuropathic pain rats in development, with 30 days old in the beginning of experiments. Eight-five male *Wistar* rats were subjected to chronic constriction injury. After establishment of NP, bimodal tDCS was applied to the rats for eight consecutive days, for 20 minutes each session. Subsequently, nociceptive behavior was assessed at baseline, 14 days after surgery, 1 day and 7 days after the end of tDCS. The rats were sacrificed 8 days after the last session of tDCS. An increase in the nociceptive threshold was observed in rats in development 1 day after the end of tDCS (short-term effect), but this effect was not maintained 7 days after the end of tDCS (long-term effect). Furthermore, brain derived neurotrophic factor (BDNF) levels were analyzed in the frontal cortex, spinal cord and serum using ELISA assays. The neuropathic pain model showed an effect of BDNF in the spinal cord of rats in development. There were no effects of BNDF levels of pain or tDCS in the frontal cortex or serum. In conclusion, tDCS is an effective technique to relieve nociceptive behavior at a short-term effect in neuropathic pain rats in development, and BDNF levels were not altered at long-term effect.

## 1. Introduction

Neuropathic pain (NP) is defined as pain that arises as a direct consequence of an injury or disease that affects the somatosensory nervous system [1] due to multiple altered mechanisms, such as functional reorganization and hyperexcitability of the somatosensory and motor cortices [2]. Although uncommon, NP has been known to develop in young patients [3], and aging is an important factor for its development [4].

Additionally, several studies showed that the application of weak electrical currents to the cortex alleviates neuropathic pain symptoms [2],[5]. Transcranial direct current stimulation (tDCS) is a noninvasive, safe and well-tolerated technique [6],[7], consisting of the application of a weak electric current to the cortex [8]. tDCS has been shown to modify cortical excitability because it provides sufficient electrical current to the cortical and subcortical areas [8]–[10] and its effects have been described on NP in clinical [11] and preclinical studies [2],[12].

Furthermore, several studies have shown that tDCS applied to the primary motor cortex or frontal cortex significantly reduces pain [13]. Some studies have described that the analgesic effect of tDCS is associated with a reduction in interleukin (IL)-1β and IL-10 levels in the spinal cord [2] and a decrease in brain-derived neurotrophic factor (BDNF) levels in the spinal cord and brainstem [14]. BDNF plays a crucial role in neuroplasticity [15] and is also related to the effects of tDCS during NP in some central nervous structures [16]–[18]. In addition, BNDF plays an important role in the development [19]. Low rates of age-related BDNF secretion can lead to synaptic connectivity alterations and to degeneration [20].

The effects of tDCS in the BDNF levels were describe in adult and/or older rats with neuropathic pain (55 to 65-day-old or (200–250 g or 260–320 g) in the beginning of experiments [12],[21]–[23] and with inflammatory pain, in a previous study of our group [24]. However, despite of our knowledge, this is the first study that aimed to understand the effects of tDCS in rats in development (30-day-old (75–100 g) in the beginning of experiment) induced with NP through chronic constriction injury by evaluating nociceptive behaviour using the von Frey test and BDNF levels in the CNS structures and serum. In this study, we hypothesize that rats in development with neuropathic pain will develop decreased nociceptive threshold and that tDCS treatment will increase the nociceptive threshold, at short and long-term effect, mediated by a reduction in the BDNF levels.

## 2. Methods

### 2.1. Animals

Eighty-five male *Wistar* rats aged 30-day (75–100 g) were provided by the Central Vivarium of Federal University of Pelotas (UFPel). Before the experiments, the rats were acclimatized to the maintenance room for 4 days until they reached 30-day-old. They were randomly grouped by weight into three main groups (control, sham-lesion (SL) and NP) and kept in three to four in cages covered with wood shavings (65 × 25 × 15 cm). The animals were kept in a controlled environment with a light-dark cycle of 12 h at 22 ± 2 °C with water and food *ad libitum*. The Ethics Committee on Animal Experimentation approved all experiments and procedures (CEEA #10480-2014) that were performed following the Guide for Care and Use of Laboratory Animals, the Brazilian law 11.794/08 and ARRIVE guidelines, which establishes procedures for the scientific use of animals.

### 2.2. Mechanical hypersensitivity test

An automatic von Frey anaesthesiometer (Insight, São Paulo, Brazil) was used to detect nociceptive threshold. A day prior to the test, the rats were placed in the apparatus made of polypropylene cages (12 × 20 × 17 cm) and habituated for 20 min [25]. The test consists of applying force to the right paw of the rats to detect the threshold at which the paw was withdrawn, which is defined as nociceptive threshold. Pressure intensity was automatically recorded after paw withdrawal [26]. To the test, the threshold of each rat was verified three times and recorded in grams, and the average was used as a behavioral response.

### 2.3. Neuropathic pain model

Before NP induction, all rats were subjected to the von Frey test (baseline) to evaluate the homogeneity of nociceptive threshold. To induce NP, chronic constriction injury (CCI) of the sciatic nerve was used according to the model described by Bennett and Xie [27]. Briefly, rats were anesthetized with ketamine (80 mg/kg) and xylazine (10 mg/kg). The right leg was shaved in the procedure region, and skin antisepsis was performed using 2% iodine-alcohol. An incision was then made to expose the sciatic nerve, and three ligatures without impeding epineural blood flow were tied (Chromic Catgut 4.0) at 1 mm intervals. Only one researcher executed the ligatures to ensure equal constriction in all rats. Subsequently, the skin was sutured with Mononylon 4.0. For sham surgery, the sciatic nerve was exposed during the procedure, but the nerve was not ligated. Rats in the control group were not subjected to any surgical procedure. All animals that underwent the surgical procedure received analgesia by subcutaneous injection of 10 mg/kg of tramadol [4] immediately at the end of the surgery.

### 2.4. Experimental procedures and tDCS

**Figure 1.**
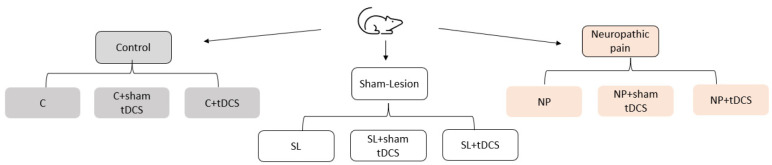
Groups of research.

**Figure 2.**
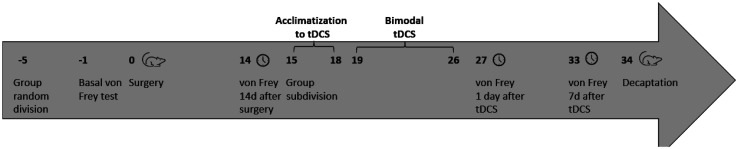
Timeline of experiments.

NP establishment was confirmed by a nociceptive test 14-day after surgery. Rats were then subdivided into the following subgroups: Control (C); control + sham-tDCS (C + sham-tDCS); control + tDCS (C + tDCS); SL; SL + sham-tDCS; SL + tDCS; NP; NP + sham-tDCS; and NP + tDCS (Figure 1). The rats were then subjected to tDCS treatment. tDCS consisted of a 20-min session of bimodal tDCS for 8 days, as described by Lopes et al. [22] and Santos et al. [28]. The battery-powered stimulator emitted a constant direct current of 0.5 mA to the electrodes. To ensure the adherence of the electrodes, the heads of CCI and sham rats were shaved. For sham stimulation, the electrodes were placed in the same position as the active tDCS, but the electrodes were turned off. Control rats were not subjected to any procedure. Subsequently, nociceptive threshold was verified 1 day after the last tDCS session (short-term effect) and 7 days after the last tDCS session (long-term effect). Eight days after the end of tDCS, the rats were killed by decaptation, and the CNS structures and total blood were collected (Figure 2). The frontal cortex, spinal cord and total blood samples were collected for biochemical analyses. Total blood was centrifuged at 3000 g for 15 min to obtain serum. All structures were kept frozen at –80 °C until processing.

### 2.5. BDNF assays

Only one researcher executed the biochemical analyses, and the groups of research were blinded for this one person. BDNF levels were measured using a sandwich ELISA with monoclonal antibodies specific for BDNF (R&D Systems, Minneapolis, MN, USA). Bradford's method was used to determine the total protein, using bovine serum albumin as the standard [29]. Results are expressed as pg/mg protein for central nervous structures and as ng/ml for serum.

### 2.6. Statistical analyses

A generalized estimating equation followed by a Bonferroni correction was used to analyze the results of the behavioral tests, considering pain measured at different times and tDCS as independent variables. For biochemical analyses, a two-way analysis of variance followed by Tukey was performed to compare the biochemical data between groups, considering pain and tDCS as independent variables. Data were expressed as the mean ± standard deviation of the mean (S.D.) using an alpha of 5% and considered significant at P ≤ 0.05. SPSS (version 20.0) for Windows was used to process results from behavioral tests, and GraphPad Prism 8.4.3 was used to perform results from biochemical analyses.

## 3. Results

### 3.1. Nociceptive threshold

**Figure 3.**
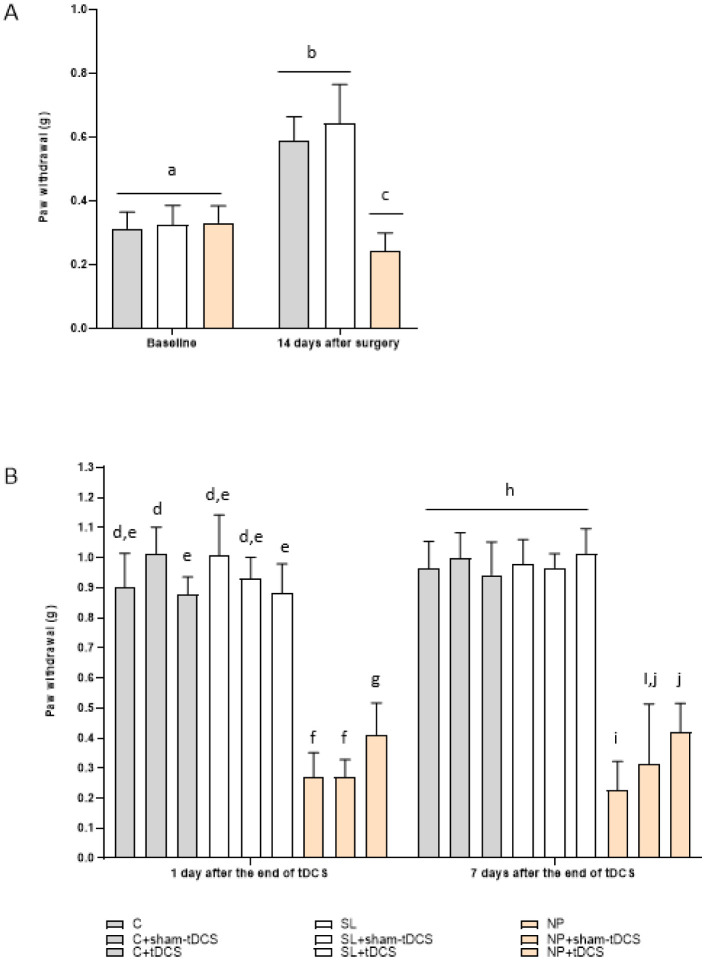
Effect of tDCS on the nociceptive threshold response by the von Frey electronics test of rats in development. Control (C); Control + sham tDCS (C + sham-tDCS); Control + tDCS (C + tDCS); Sham Lesion (SL); Sham lesion + sham-tDCS (SL + sham tDCS); Sham lesion + tDCS (SL + tDCS); Neuropathic pain (NP); Neuropathic pain + sham-tDCS (NP + sham-tDCS); Neuropathic pain + tDCS (NP + tDCS). Data are presented as the mean ± SD, (n = 7–11). Different letter subscripts (a through j) indicate a statistically significant difference between the groups. **Panel A:** Threshold at baseline and 14 days after surgery C (n = 30), SL (n = 25), NP (n = 30). **Panel B:** Effects of tDCS 1 day and 7 days after the end of treatment (GEE/Bonferroni Interaction time x group (Wald χ^2^ = 1,709.97; 24, P < 0.001, n = 7–11; C (n = 10), C + sham-tDCS (n = 10), C + tDCS (n = 10), SL (n = 7), SL + sham-tDCS (n = 9), SL + tDCS (n = 9), NP (n = 9), NP + sham-tDCS (n = 10), NP + tDCS (n = 11)).

Regarding the nociceptive behavior test, the generalized estimation equation presented a time effect (Wald χ^2^ = 2.010.04; 3, P < 0.001) characterized by a significative difference between basal, 14 days after surgery, 1 day and 7 days after the end of tDCS verifications, except comparing 1 day and 7 days after tDCS treatment (P = 0.191). A group effect (Wald χ^2^ = 1.455.90; 8, P < 0.001) was observed comparing NP groups (exposed to the CCI model) with the C and SL groups (P < 0.0001) and with NP + tDCS group (P = 0.007). Additionally, it was observed an interaction time x group (Wald χ^2^ = 1.709.97; 24, P < 0.001). At baseline, there were no significant differences between the groups (Wald χ^2^ = 12.03; 8, P = 0.150). At 14-day after surgery, the rats demonstrated increased threshold in the paw withdrawal test compared to the baseline measure, since they are in development (P < 0.001) and the three NP groups showed a significant increase in the nociceptive behavior (lower threshold) compared to all C and all SL groups (P < 0.001) (Figure 3, panel A). The treated rats (NP + tDCS) showed a significant increase in the nociceptive threshold at 1 day after the end of treatment compared to NP (P = 0.012) and NP + sham-tDCS (P = 0.002) groups, and NP showed a similar effect of NP + sham-tDCS group (P > 0.05), suggesting an anti-hyperalgesic effect. In addition, it was observed a decreased effect of C + tDCS (P = 0.001) and SL + tDCS (P = 0.039) compared to C + sham-tDCS (Wald χ^2^ = 1.429.56; 8, P < 0.001). However, 7-day after the end of tDCS, NP + tDCS group showed significant increase compared to NP group (P < 0.001), but this effect was similar to the sham effect (NP + sham-tDCS, P > 0.05) (Wald χ^2^ = 928.06; 24, P < 0.001) (Figure 3, panel B).

### 3.2. BDNF analyses

**Figure 4.**
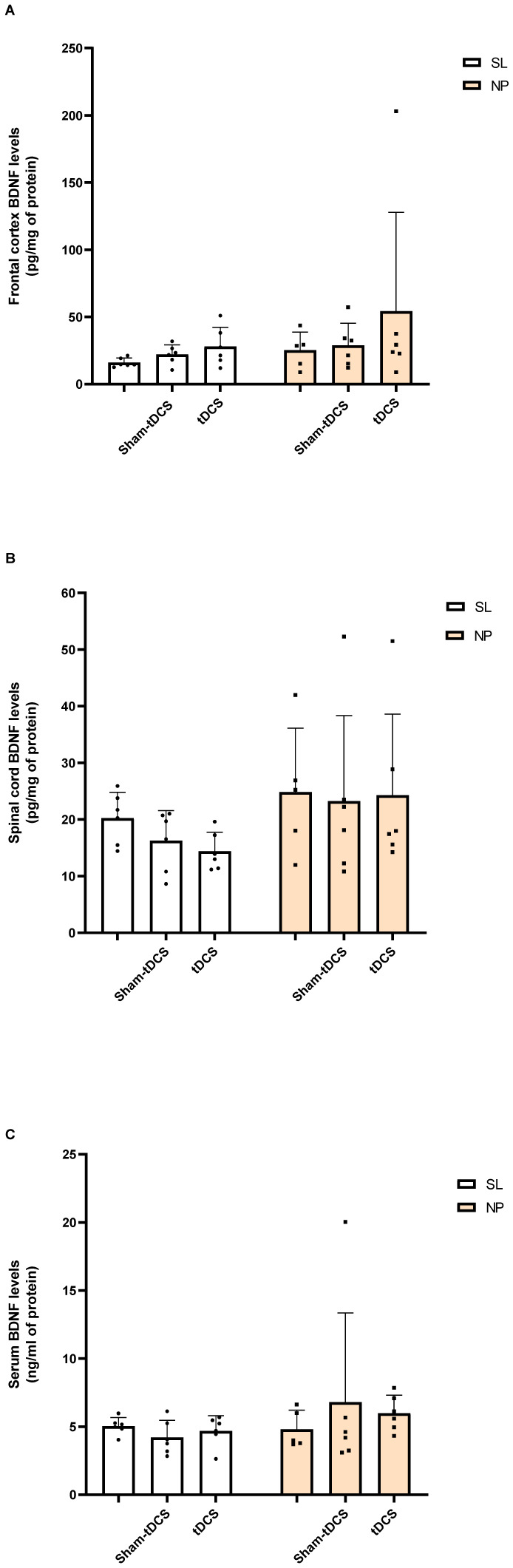
BDNF levels of rats in development at the long-term effect. Sham Lesion (SL); Sham lesion + sham-tDCS (SL + sham tDCS); Sham lesion + tDCS (SL + tDCS); Neuropathic pain (NP); Neuropathic pain + sham-tDCS (NP + sham-tDCS); Neuropathic pain + tDCS (NP + tDCS). Data are presented as the mean ± SD, (n = 5–6). **Panel A: Frontal cortex BDNF levels:** There were no effects of pain (F_(1,29)_ = 1.635, P = 0.211) or tDCS F_(2,29)_ = 1.272, P = 0.295) (Two-way ANOVA/Tukey, n = 5–6). **Panel B: Spinal cord BDNF levels:** There was an effect of pain (F_(1,29)_ = 4.322 P = 0.0466). tDCS effect was not statistically significative different between groups (F_(2,29)_ = 0.3370, P = 0.7167) and no interaction pain x tDCS was found (F_(2,29)_ = 0.1971, P = 0.8222) (Two-way ANOVA/Tukey, n = 5–6). **Panel C: Serum BDNF levels:** there were no differences between groups for pain (F_(1,29)_ = 1.530, P = 0.2260) or tDCS (F_(2,29)_ = 0.1159, P = 0.8910) (Two-way ANOVA/Tukey, n = 5–6).

For biochemical analyses, only SL and NP rats were included, as the control and sham groups did not show differences in nociceptive threshold at the 7 days after tDCS treatment according to the von Frey test (a day prior rats being sacrificed). In the frontal cortex of rats in development exposed to the pain model BDNF levels did not show a statistically significant difference between the groups for pain (F_(1,29)_ = 1.635, P = 0.211) or tDCS (F_(2,29)_ = 1.272, P = 0.295), indicating that pain and tDCS treatment could not modify frontal cortex BDNF levels in the long term (Figure 4, Panel A).

Moreover, in the spinal cord, an effect of pain was observed (F_(1,29)_ = 4.322, P = 0.0466) in BDNF levels, but no effect was detected in the post hoc test. Furthermore, there was no effect of tDCS in BDNF levels regarding spinal cord analyses (F_(2,29)_ = 0.3370, P = 0.7167) or interaction pain x tDCS (F_(2,29)_ = 0.1971, P = 0.8222) (Figure 4, Panel B). BDNF serum levels were not modified by the pain model (F_(1,29)_ = 1.530, P = 0.2260) or tDCS treatment (F_(2,29)_ = 0.1159, P = 0.8910) (Figure 4, Panel C).

## 4. Discussion

Here, we hypothesize that rats in development with neuropathic pain will develop decreased nociceptive threshold, and that tDCS treatment will increase the nociceptive threshold, at short and long-term effect, mediated by a reduction in the BDNF levels in rats with chronic constriction injury.

Regarding nociceptive behaviour, at the baseline, rats presented a similar effect, characterizing the homogeneity of the nociceptive threshold. Fourteen days after NP induction, the rats demonstrated increased in the paw withdrawal compared to the baseline measure. It is important to note that the rats were 30-day-old at the induction of CCI and 64-day-old at the end of experiments. Our results show an increase of the nociceptive threshold according to the development of rats, corroborating Nunes et al. [30]. Furthermore, all rats subjected to CCI surgery showed increased nociceptive threshold, as the threshold increased significantly in rats in development. The test of hypersensitivity to mechanical stimuli is the most common method of measuring the magnification of a neuropathological condition [31]. Considering results obtained for CCI group, mechanical allodynia and hyperalgesia are behavioral signs of NP [32].

A study conducted by Howard et al. [33] investigated the effects of neuropathic pain in different developmental ages using the spared nerve injury (SNI) model. The authors investigated the effects of SNI at the 3-, 10-, 21- and 33-days old rats for 28 days, observing nociceptive behavior at 7, 14 and 28 days after surgery. They found no differences in the threshold in rats of 3 days. For rats of 10 and 21 days they found a decreased threshold in the SNI rats compared to control group or contralateral paw, but the effects were not maintained for 14 and 28 days. At 33 days old they found decreased threshold at the 7, 14 and 28 days after surgery. In this same work the authors evaluated the effects of NP using CCI model in rats aged at 10 and 60 days. The authors found no differences in the threshold of 10 days old rats at any point observed (7, 14 and 28 days after surgery). At 60 days, old rats with CCI showed a significative difference was observed 7, 14 and 28 days after surgery, compared with contralateral paw. We demonstrate the influence of age in nociceptive behavior of NP development.

In addition, our results corroborate previous studies using the CCI model in *Wistar* rats aged 55–65 days [2] and 8 weeks and 60 days [22],[28]. The CCI model is the most used method in peripheral nerve injury NP experiments. It can produce significant and stable pain hypersensitivity for at least 1-month after NP induction. This model is widely used to investigate the pathophysiological mechanisms and potential therapeutic agents for the treatment of NP [4].

Interestingly, bimodal tDCS was able to promote hyper nociceptive behavior 1day after the last session, but at 7 days after the last session, the analgesic effect was lost in rats in development, demonstrating just a short-term effect of treatment. These effects show the possible effects of manipulation and/or immobilization in the sham-tDCS groups, which characterizes a limitation of our study. In contrast, in our previous study, we demonstrate the effects of bimodal tDCS in neuropathic pain rats at 60 days old at long term-effect (7 days after the last session of tDCS), but this effect was not observed at the short-term effect (24 hours after the last session of tDCS) [34]. Then, we can suggest different responses to the tDCS treatment in different ages. Other studies have shown that tDCS can reverse hyper nociceptive behavior at the long-term effects of tDCS in adult rats using similar tDCS protocol and NP model [2],[22].

Additionally, it was observed a decreased nociceptive threshold in rats of the C and SL groups that received tDCS treatment compared to rats of the C group that received sham stimulation, demonstrating an effect of tDCS treatment in rats without CCI, but these results were not maintained at long term-effect. In this study, we showed the effects of tDCS in rats in development with NP. In addition, age can significantly influence the development of NP after CCI induction [4]. According to studies with 40 healthy adult humans (median, 68 years) and young adults (median, 23 years), older adults exhibited a delayed response to anodal tDCS compared with young adults [15]. The induced after-effects of tDCS depend on the polarity, duration and stimulation intensity [35]. Studies have shown that anodal tDCS increases the corticospinal excitability of local stimulation and distant areas of the stimulation site, attributed to interconnections between these sites [36]. The full mechanism of action of tDCS is not well understood; however, evidence suggests that tDCS can act through direct and indirect pathways [37]. We used bimodal stimulation, and BDNF levels in rats in development in the frontal cortex, spinal cord and serum were assessed.

At 30 days with neuropathic pain, BDNF levels in the frontal cortex of the NP group were not modified. This may be because the young rats demonstrated that the tDCS effects increased the threshold of NP rats (NP + tDCS group) compared with the NP and NP + sham-tDCS groups in the short term (1day after the end of tDCS), but not in the long term (7-day after the end of tDCS).

The rats were sacrificed 8 days after the end of treatment; thus, tDCS effects were not observed in these rats. For the same reason, serum BDNF levels were not altered. However, BDNF is widely distributed in the human brain, plays an important role in supporting neuronal structure and function [38] and plays various roles in development [39]. Studies by Filho et al. [12] in *Wistar* rats aged 55−65-day-old found a short-term (48-h after the end of tDCS) and a long-term (7-day after the end of tDCS) decrease in the brain stem BDNF levels, a short-term increase in spinal cord BDFN levels and a long-term decrease in serum BDNF levels in rats with NP. Corroborating, in our study, it was found a pain effect in the spinal cord BDNF levels, and comparing these analyses evaluated by Filho et al. [12] in old rats with increased nociceptive threshold by tDCS treatment with short- and long-term effects. Thus, we suggest that differences in BDNF levels in these structures should be related to age.

tDCS can induce and modulate neuroplasticity [40],[41]. Boudes and Menigoz [42] showed that BDNF mediates changes in excitatory synaptic transmission in the dorsal horn of rats with sciatic nerve injury and other studies have demonstrated the influence of this neurotrophin in NP and tDCS treatment.

Lopes et al. [21] investigated the effects of tDCS plus exercise over BDNF levels in neuropathic pain rats (280 g, approximately). The researchers evaluated the nociceptive behavior and they observed that rats exposed to CCI procedure decreased the nociceptive threshold 7 and 14 days after surgery. Thereafter, we evaluated the effects of treatment immediately, 24 hours and 7 days after the end of tDCS. The rats with neuropathic pain exposed to tDCS, exercise and tDCS plus exercise had increased nociceptive threshold immediately, 24 hours and 7 days after the end of treatment and tDCS plus exercise increased the nociceptive threshold compared to tDCS isolated, 7 days after the end of treatment. The authors observed that cortical cerebral BDNF levels were increased in rats with NP treated with tDCS, 48 h after treatment and tDCS plus exercise increased BDNF levels 7 days after the end of treatment. In the brainstem, the authors found decreased levels of BDNF in NP groups 48 hours after the end of treatment with tDCS. In the spinal cord, they found increased BDNF levels in NP group that did not receive any treatment, and 48 hours after the end of treatment, decreased BDNF levels were observed in NP group that received sham-stimulation plus exercise compared with the group with sham of NP that received sham-stimulation plus exercise.

In a similar study, Lopes et al. [22] verified the effects of tDCS associated with exercise in the BDNF levels in rats (8 weeks old) with neuropathic pain. The authors observed increased hippocampal BDNF levels occasioned by tDCS plus exercise in NP groups, at the short-term. Long-term, they found that tDCS and exercise prevented a reduction in BDNF levels in the hippocampus. In the sciatic nerve. Short-term, it was observed increased BDNF levels in NP groups by the effects of tDCS and exercise.

We found an effect of pain in the spinal cord BDNF levels in rats in development. For cortical and serum analyzes, no effects were found in this study long-term; however, the effects of tDCS were well described in other studies with adult rats, showing its influence in NP and its modulation by tDCS treatment.

Our findings add new information since tDCS effectively reduces hyper nociceptive behavior in rats in development with CCI injury, showing that tDCS can reduce hyperalgesia at this phase of development in the short-term effect. Biochemical analyses of BDNF levels in rats in development were insufficient to elucidate the effects of tDCS on neuroplasticity because it was evaluated only in the long term, and more studies are warranted to evaluate short-term BDNF levels in rats in development. We believe that BDNF will be modified by tDCS treatment at this time point for young neuropathic pain rats, in different nervous systems structures.

It is relevant to know the effects of tDCS and intermediates of NP in the phase of development since age can influence the levels of these intermediates (biomarkers and immunity cells), and consequently the response to the treatment in young people. Although NP occurs less frequently at the early age, it could happen and need of another kind of intervention them the used in old patients.

This study contributes to understand the effects of tDCS in the nociceptive behavior and BDNF levels of neuropathic pain rats in development. Furthermore, the NP and tDCS effects in the nociceptive behaviour and in BDNF levels of rats in development was discussed using previous published studies conducted with adult/older rats with NP treated with tDCS and that evaluate the influence of BDNF levels and other biomarkers in NP and tDCS treatment. Despite that, further studies are necessary to elucidate the effects of BDNF and tDCS on NP at the early age.

Finally, our study had some limitations, including that we used only male rats; we used the whole spinal cord for analyses, but different regions of the spinal cord could reflect in different levels of intermediates of NP; BDNF levels were evaluated only at long-term, but important modifications could occur short-term effect since a tDCS effect was observed in the nociceptive threshold just at this time point; and it was observed a sham-effect occasioned by manipulation and/or immobilization of rats, as it was previously mentioned in this discussion, so a different form of immobilization of rats should be considered for future studies.

## 5. Conclusion

Analyzing these data made it possible to conclude that NP was induced 14-day after CCI, and tDCS promoted relief of hyperalgesic behavior, in the short term, in 30-day-old rats. Additionally, it was observed an effect of pain in the spinal cord BDNF levels; however, this effect was not confirmed in the post hoc test. The frontal cortex and serum BDNF levels were not altered by pain or long-term tDCS. In conclusion, these results bring the novelty of behavioral and biochemical responses to the tDCS effects in neuropathic pain rats in development; however, more studies are necessary to elucidate the effects of tDCS in the BDNF levels in rats in development with NP at the short-term.

## 6. Future directions

For future research, we recommend using another kind of immobilization of rats for tDCS application, since immobilization used in this experiment showed to influence some parameters analyzed. Furthermore, new studies about the effects of tDCS in neuropathic pain rats in development are necessary to evaluate other intermediates/biomarkers associated with the early age nociceptive responses.

## Ethics approval

The manuscript does not contain clinical studies or patient data.

This study was performed in accordance with Brazilian law 11,794. Approval was granted by the Comitê de Ética em Experimentação Animal (CEEA 10480-2014) of the Universidade Federal de Pelotas by the Veterinary Medicine Dra. Anelize de Oliveira Campello Felix on July 20, 2015.

## Funding

This study was financed in part by the Agência Federal Brasileira para Coordenação de Aperfeiçoamento de Pessoal de Nível Superior (CAPES)-MD - Finance Code 001 (P.C. Crespo), and the Conselho Nacional de Desenvolvimento Científico e Tecnológico (CNPq) - MCTI/CNPQ/Universal 14/2014 (Izabel Cristina Custodio de Souza).

## References

[1] (2014). International association for the study of pain. What is neuropathic pain?. Global year against neuropathic pain, USA.

[2] Cioato SG, Medeiros LF, Marques Filho PR (2016). Long-Lasting Effect of Transcranial Direct Current Stimulation in the Reversal of Hyperalgesia and Cytokine Alterations Induced by the Neuropathic Pain Model. Brain Stimul.

[3] Vega-Avelaira D, Géranton SM, Fitzgerald M (2009). Differential regulation of immune responses and macrophage/neuron interactions in the dorsal root ganglion in young and adult rats following nerve injury. Mol Pain.

[4] Austin PJ, Wu A, Moalem-Taylor G (2012). Chronic constriction of the sciatic nerve and pain hypersensitivity testing in rats. J Vis Exp.

[5] Boldt I, Eriks-Hoogland I, Brinkhof MW (2014). Non-pharmacological interventions for chronic pain in people with spinal cord injury. Cochrane Database Syst Rev.

[6] Antal A, Terney D, Kühnl S (2010). Anodal Transcranial Direct Current Stimulation of the Motor Cortex Ameliorates Chronic Pain and Reduces Short Intracortical Inhibition. J Pain Symptom Manage.

[7] Liu A, Bryant A, Jefferson A (2016). Exploring the efficacy of a 5-day course of transcranial direct current stimulation (TDCS) on depression and memory function in patients with well-controlled temporal lobe epilepsy. Epilepsy Behav.

[8] Dimov LF, Franciosi AC, Pinheiro Campos AC (2016). Top-Down Effect of Direct Current Stimulation on the Nociceptive Response of Rats. PLoS One.

[9] Roche N, Lackmy A, Achache V (2011). Effects of anodal transcranial direct current stimulation over the leg motor area on lumbar spinal network excitability in healthy subjects. J Physiol.

[10] Schestatsky P, Morales-Quezada L, Fregni F (2013). Simultaneous EEG monitoring during transcranial direct current stimulation. J Vis Exp.

[11] Palm U, Chalah MA, Padberg F (2016). Effects of transcranial random noise stimulation (tRNS) on affect, pain and attention in multiple sclerosis. Restor Neurol Neurosci.

[12] Filho PRM, Vercelino R, Cioato SG (2016). Transcranial direct current stimulation (tDCS) reverts behavioral alterations and brainstem BDNF level increase induced by neuropathic pain model: Long-lasting effect. Prog Neuro-Psychopharmacology Biol Psychiatry.

[13] DaSilva AF, Volz MS, Bikson M (2011). Electrode positioning and montage in transcranial direct current stimulation. J Vis Exp.

[14] Spezia Adachi LN, Caumo W, Laste G (2012). Reversal of chronic stress-induced pain by transcranial direct current stimulation (tDCS) in an animal model. Brain Res.

[15] Fujiyama H, Hyde J, Hinder MR (2014). Delayed plastic responses to anodal tDCS in older adults. Front Aging Neurosci.

[16] Ayache SS, Palm U, Chalah MA (2016). Prefrontal tDCS decreases pain in patients with multiple sclerosis. Front Neurosci.

[17] Knotkova H, Portenoy RK, Cruciani RA (2013). Transcranial direct current stimulation (tDCS) relieved itching in a patient with chronic neuropathic pain. Clin J Pain.

[18] Volz MS, Farmer A, Siegmund B (2016). Reduction of chronic abdominal pain in patients with inflammatory bowel disease through transcranial direct current stimulation: a randomized controlled. Pain.

[19] Khan N, Smith MT (2015). Neurotrophins and neuropathic pain: Role in pathobiology. Molecules.

[20] Oh H, Lewis DA, Sibille E (2016). The Role of BDNF in Age-Dependent Changes of Excitatory and Inhibitory Synaptic Markers in the Human Prefrontal Cortex. Neuropsychopharmacology.

[21] Lopes BC, Medeiros LF, Silva de Souza V (2020). Transcranial direct current stimulation combined with exercise modulates the inflammatory profile and hyperalgesic response in rats subjected to a neuropathic pain model: Long-term effects. Brain Stimul.

[22] Lopes BC, Medeiros LF, Stein DJ (2021). tDCS and exercise improve anxiety-like behavior and locomotion in chronic pain rats via modulation of neurotrophins and inflammatory mediators. Behav Brain Res.

[23] Santos DS, Medeiros LF, Stein DJ (2021). Bimodal transcranial direct current stimulation reduces alcohol consumption and induces long-term neurochemical changes in rats with neuropathic pain. Neurosci Lett.

[24] Silva SP, Martins OG, Medeiros LF (2022). Evidence of Anti-Inflammatory Effect of Transcranial Direct Current Stimulation in a CFA-Induced Chronic Inflammatory Pain Model in Wistar Rats. Neuroimmunomodulation.

[25] Netto CA, Siegfried B, Izquierdo I (1987). Analgesia induced by exposure to a novel environment in rats: Effect of concurrent and post-training stressful stimulation. Behav Neural Biol.

[26] Vivancos GG, Verri WA, Cunha TM (2004). An electronic pressure-meter nociception paw test for rats. Brazilian J Med Biol Res.

[27] Bennett GJ, Xie YK (1988). A peripheral mononeuropathy in rat that produces disorders of pain sensation like those seen in man. Pain.

[28] Santos DS, Lopes BC, Medeiros LF (2020). Transcranial Direct Current Stimulation (tDCS) Induces Analgesia in Rats with Neuropathic Pain and Alcohol Abstinence. Neurochem Res.

[29] Bradford MM (1976). A rapid and sensitive method for the quantitation of microgram quantities of protein utilizing the principle of protein-dye binding. Anal Biochem.

[30] Nunes EA, Medeiros LF, de Freitas JS (2016). Morphine Exposure during Early Life Alters Thermal and Mechanical Thresholds in Rats. Int J Dev Neurosci.

[31] Boada MD, Gutierrez S, Aschenbrenner CA (2015). Nerve injury induces a new profile of tactile and mechanical nociceptor input from undamaged peripheral afferents. J Neurophysiol.

[32] Lu VB, Biggs JE, Stebbing MJ (2009). Brain-derived neurotrophic factor drives the changes in excitatory synaptic transmission in the rat superficial dorsal horn that follow sciatic nerve injury. J Physiol.

[33] Howard RF, Walker SM, Mota MP (2005). The ontogeny of neuropathic pain: postnatal onset of mechanical allodynia in rat spared nerve injury (SNI) and chronic constriction injury (CCI) models. Pain.

[34] Centeno Crespo P, Anderson Meira Martins L, Camacho Dos Reis C (2023). Transcranial direct current stimulation effects in the pain threshold and in oxidative stress parameters of neuropathic pain rats. Neurosci Lett.

[35] Csifcsak G, Antal A, Hillers F (2009). Modulatory effects of transcranial direct current stimulation on laser-evoked potentials. Pain Med.

[36] Vaseghi B, Zoghi M, Jaberzadeh S (2015). The effects of anodal-tDCS on corticospinal excitability enhancement and its after-effects: conventional vs. unihemispheric concurrent dual-site stimulation. Front Hum Neurosci.

[37] Coull JA, Beggs S, Boudreau D (2005). BDNF from microglia causes the shift in neuronal anion gradient underlying neuropathic pain. Nature.

[38] Soltész F, Suckling J, Lawrence P (2014). Identification of BDNF sensitive electrophysiological markers of synaptic activity and their structural correlates in healthy subjects using a genetic approach utilizing the functional BDNF Val66Met polymorphism. PLoS One.

[39] Cheeran B, Talelli P, Mori F (2008). A common polymorphism in the brain derived neurotrophic factor gene (BDNF) modulates human cortical plasticity and the response to rTMS. J Physiol.

[40] Kuo MF, Paulus W, Nitsche MA (2014). Therapeutic effects of non-invasive brain stimulation with direct currents (tDCS) in neuropsychiatric diseases. Neuroimage.

[41] Pirulli C, Fertonani A, Miniussi C (2013). The role of timing in the induction of neuromodulation in perceptual learning by transcranial electric stimulation. Brain Stimul.

[42] Boudes M, Menigoz A (2009). Non-neuronal BDNF, a key player in development of central sensitization and neuropathic pain. J Physiol.

